# Dexamethasone Admixture for Hypersensitivity Prevention in Botulinum Toxin Type A Treatment: A Case Report

**DOI:** 10.1111/jocd.70837

**Published:** 2026-04-02

**Authors:** Ximeng Jia, Yutong Liang, Qi Chen, Jintian Hu

**Affiliations:** ^1^ Laser Aesthetic Center Plastic Surgery Hospital, Chinese Academy of Medical Sciences and Peking Union Medical College Beijing China; ^2^ Department of Cosmetic Injection Center Plastic Surgery Hospital, Chinese Academy of Medical Sciences and Peking Union Medical College Beijing China; ^3^ Second Clinical Medical School of Medicine Xinjiang Medical University Xinjiang People's Republic of China

## 1 Introduction

An allergic reaction is an abnormal physiological response of the immune system to harmless substances, essentially mediated by IgE antibodies as a type I hypersensitivity reaction. Local allergic reactions may manifest as symptoms such as redness, swelling, itching, pain, and rashes, typically appearing shortly after injection. While most cases are mild and self‐limiting, they may cause significant discomfort in some patients and even affect treatment efficacy. Oral antihistamines (e.g., loratadine) or topical steroids (e.g., tacrolimus) are commonly used to alleviate local allergic symptoms [1, 2, 3]. Botulinum toxin type A (BoNT‐A) is generally well‐tolerated, with rare allergic reactions [4, 5, 6, 7]. The few reported cases of post‐botulinum toxin injection allergies have all occurred in the facial region, making this study the first documented case of a local allergic reaction following botulinum toxin injection in the neck area. Meanwhile, we have innovatively proposed a new safe and effective preventive solution.

## 2 Case Report

**FIGURE 1:**
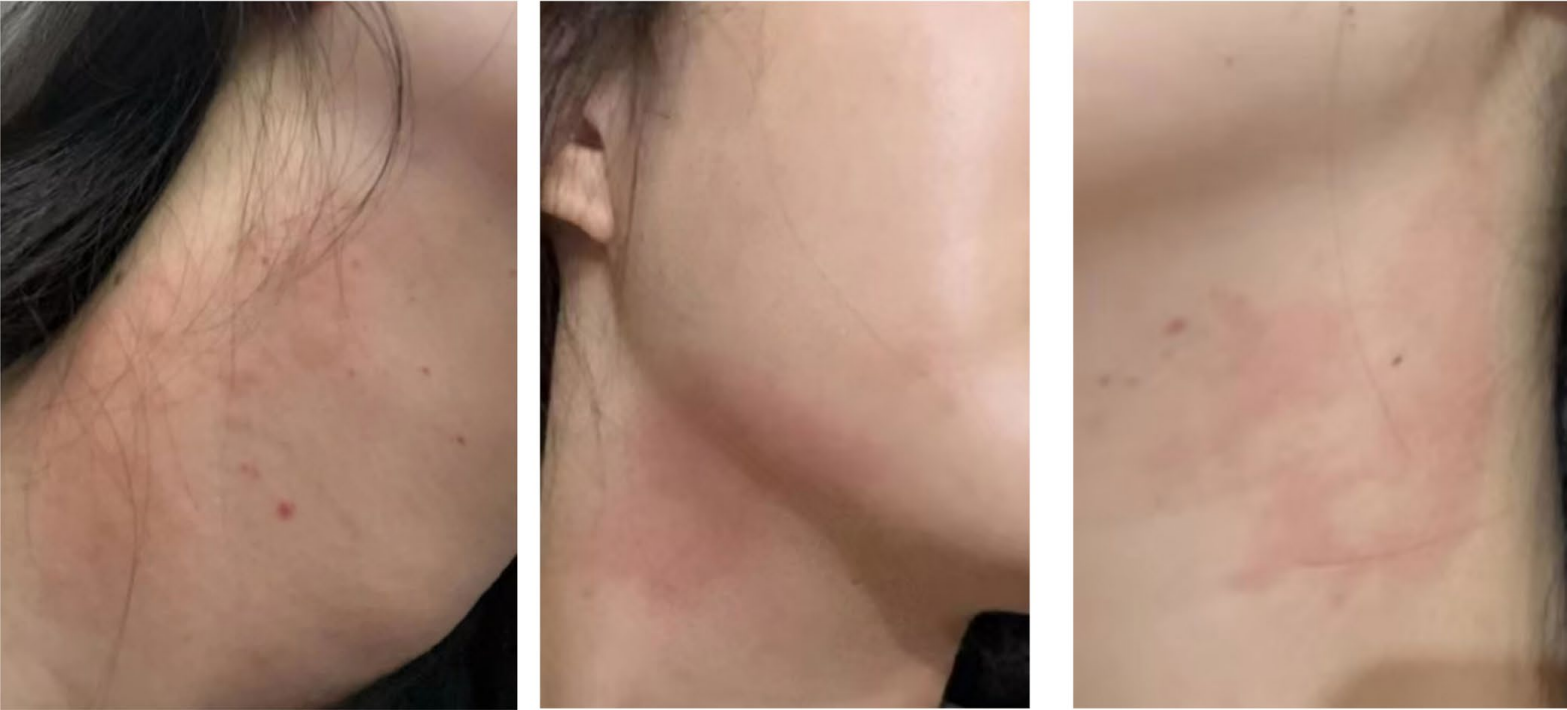
Clinical photograph taken 2 h after the first BoNT‐A injection, showing localized erythema, mild swelling, and a patchy rash over the bilateral mandibular and cervical injection areas.

**FIGURE 2:**
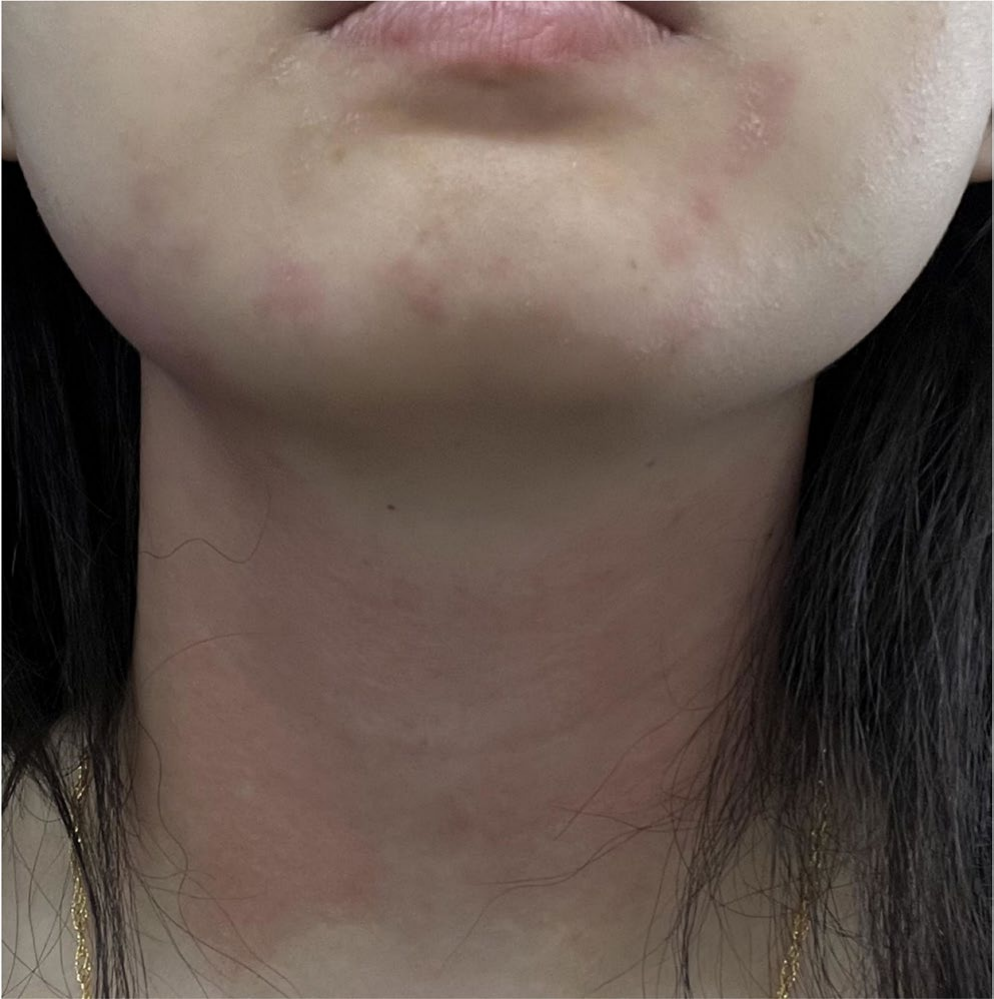
Clinical photograph taken 2 h after the second BoNT‐A injection, again demonstrating localized erythema and mild swelling confined to the same bilateral mandibular and cervical regions, consistent with the prior reaction.

A 25‐year‐old woman presented for jawline enhancement to improve lower facial contouring. She had no significant medical history, was taking no regular medications, and had not received botulinum toxin injections prior to the treatments described. For each of the initial two treatments, one vial of Botulinum Toxin Type A (Botox; manufactured by Allergan Pharmaceuticals Ireland, Westport, County Mayo, Ireland; 100 U) was utilized. This product has a shelf life of 1 to 3 years, necessitates refrigeration at temperatures between 2°C and 8°C, should be used immediately after opening, and repeated extraction should be avoided to prevent contamination. After reconstituting with 2 mL of normal saline, intramuscular injections were administered to the bilateral platysmal bands using a 30‐gauge needle (Supplementary Figure 1). Approximately 2 h after each injection, the patient developed pronounced erythema in the bilateral mandibular and cervical injection areas (approximately 1% in extent), accompanied by swelling. The affected areas exhibited a more vivid red coloration compared to the surrounding normal skin. Upon palpation, the area exhibits warmth and is associated with moderate‐to‐severe pruritus (Figure 1). There are no accompanying symptoms of dyspnea, dizziness, angioedema, or generalized urticaria. The lesions gradually resolved over 2–3 days without intervention. The two hypersensitivity episodes occurred 6 months apart and were identical in onset, distribution, and duration (Figure 2). Both injections were performed by the same practitioner using the same dilution and injection technique.

No formal allergy evaluation—such as serum IgE testing, skin prick testing, or patch testing—was performed as the patient declined returning to the clinic for assessment after symptom resolution on both occasions.

**FIGURE 3:**
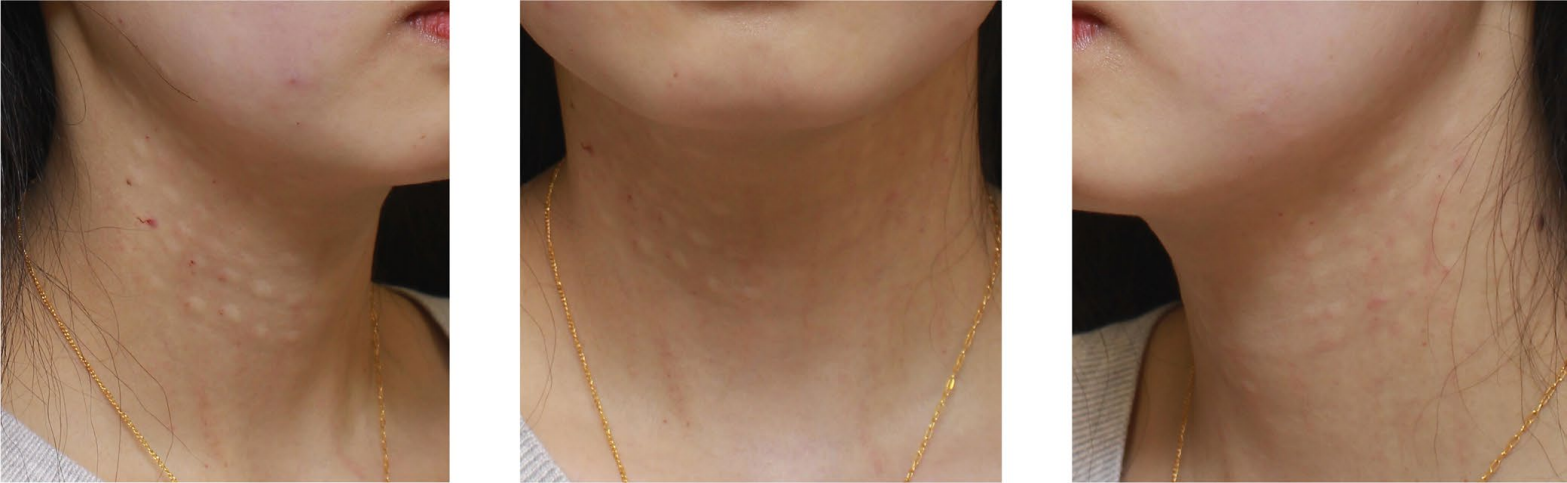
Immediate post‐treatment photograph (taken immediately after the third injection) performed with BoNT‐A diluted together with low‐dose dexamethasone, showing no visible erythema, swelling, or rash at the injection sites.

**FIGURE 4:**
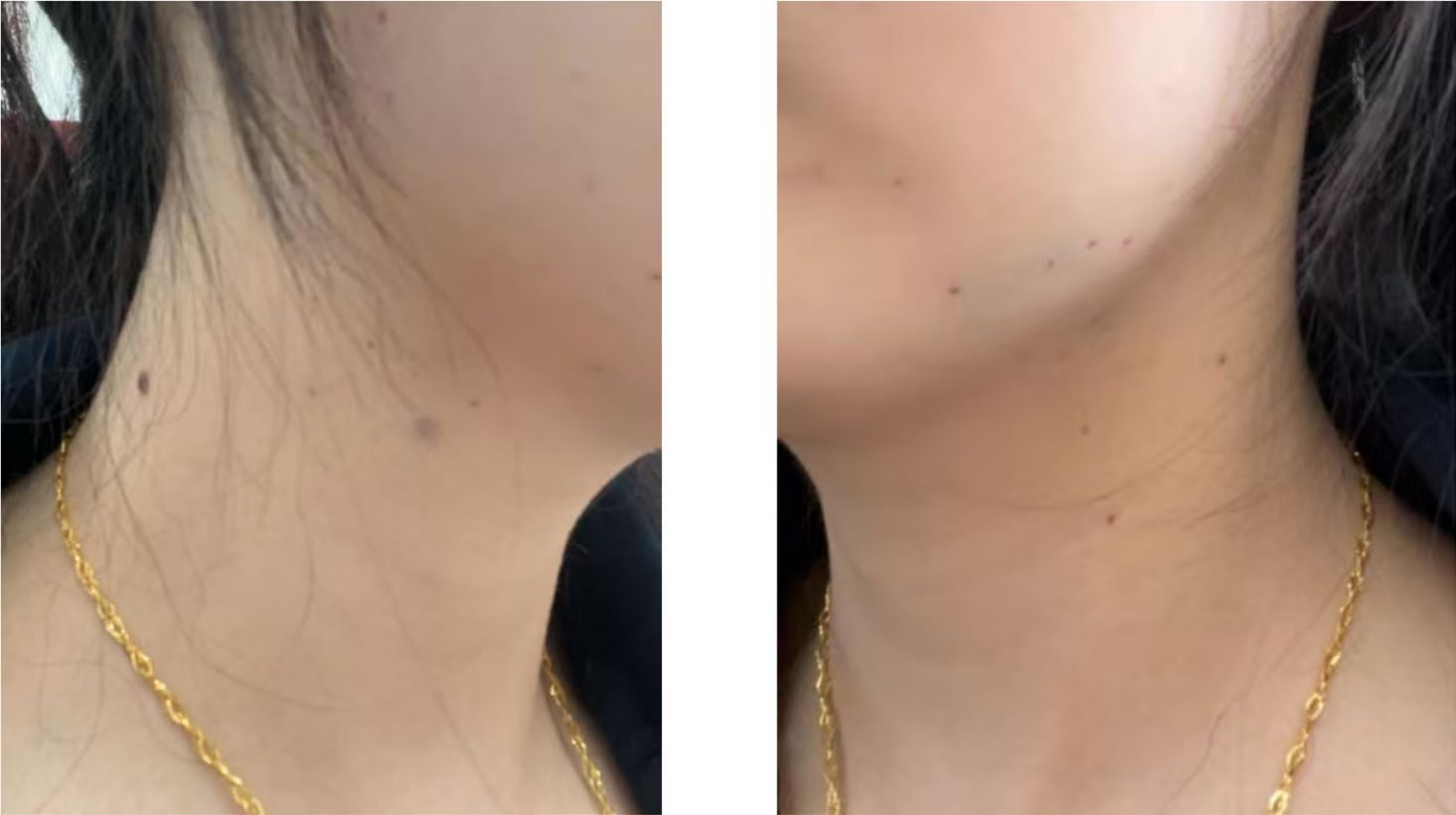
Clinical photograph taken 24 h after the third injection, demonstrating normal skin appearance without erythema, swelling, or rash in the bilateral mandibular and cervical areas.

During the third treatment session, BoNT‐A was reconstituted with 2 mL of normal saline mixed with 0.02 mg of dexamethasone (1 mg/mL; Dandong Meichen Pharmaceutical Co., approval No. H21022401) in the same syringe and injected intramuscularly into the same anatomical regions using the identical technique. No erythema, swelling, rash, or discomfort occurred intraoperatively or postoperatively. The patient tolerated the treatment well (Figure 3), and no delayed hypersensitivity reaction was observed during follow‐up. The esthetic outcome was satisfactory (Figure 4). All patients have given informed consent for the publication and use of their photographs.

## 3 Discussion

Localized cutaneous reactions following botulinum toxin type A (BoNT‐A) injection may arise from multiple mechanisms, including immunologic hypersensitivity, non‐immune irritant responses, and vascular reactivity. The patient exhibited local skin reactions approximately 2 h post‐injection on both occasions, characterized by visible blisters at the injection site. The temporal progression and clinical characteristics observed were consistent with a localized allergic reaction. The persistent manifestation of erythema and pruritic eruption after two separate treatment sessions, each lasting 2–3 days, signifies a localized hypersensitivity response rather than a mere irritant effect. Recent studies have recorded delayed‐onset plaques, urticaria‐like reactions, and secondary treatment failure subsequent to repeated esthetic BoNT‐A exposure, suggesting that both immediate and delayed immune mechanisms may contribute to injection‐site reactions [8, 9, 10].

Conversely, sensitization prior to injection typically does not account for irritating reactions at the injection site, which generally occur shortly after administration and resolve rapidly due to mechanical trauma or the pH/osmolarity of the formulation [11]. Although vascular flare remains a potential consideration, the patient's ongoing symptoms align more closely with immune‐mediated hypersensitivity, as supported by existing literature [8, 9, 10].

The reason for adding a small amount of dexamethasone to the BoNT‐A diluent is because it is known to have anti‐inflammatory and immunomodulatory effects. Glucocorticoids stop pro‐inflammatory cytokines, limit the movement of leukocytes, and lower important mediators of allergic inflammation through NF‐κB and related pathways [2, 12]. Beyond theoretical mechanisms, corticosteroid premedication has demonstrated clinical benefit in attenuating recurrent hypersensitivity reactions in high‐risk patients undergoing iodinated contrast administration [3]. Our case was about localized cutaneous hypersensitivity instead of systemic anaphylaxis, but this broader evidence supports the idea that pre‐emptive immune modulation could lower the chances of people who are already sensitized having more reactions.

Nevertheless, admixture of dexamethasone with BoNT‐A is an off‐label practice, and no studies have evaluated the physicochemical stability or diffusion characteristics of this combination. Clinical studies involving alternative diluents—such as lidocaine with or without epinephrine—suggest that BoNT‐A maintains clinical efficacy across certain pH and formulation ranges [13, 14]. However, these findings cannot be extrapolated to corticosteroid‐containing solutions. Although this study did not demonstrate differences in efficacy, alterations in the stability of modified proteins, changes in dispersion or potency, and potential local tissue effects may influence the final therapeutic outcome, either enhancing or diminishing it. The administration of a little dexamethasone dosage (0.02 mg) and the lack of side effects in this patient are encouraging; however, they are inadequate to confirm safety.

This case has inherent limitations, including the absence of formal allergy evaluation, which restricts pathophysiologic interpretation. It is noteworthy that the compatibility and long‐term safety of the dexamethasone and BoNT‐A compound preparation remain to be determined; thus, cautious consideration is still required when applying this study to clinical practice. In summary, this case illustrates a potential strategy for addressing recurrent injection‐site reactions in specific patients with a history of BoNT‐A hypersensitivity. The preliminary evidence, derived from a single observation, indicates a favorable clinical outcome, suggesting the need for further evaluation of localized low‐dose corticosteroid admixture. Further research is required to elucidate compatibility, safety, and clinical applicability.

## 4 Conclusion

This case adds to the limited literature on BoNT‐A–related hypersensitivity and highlights a potential management approach using localized low‐dose corticosteroid admixture. Further study is warranted to better define its role and clinical applicability.

## Funding

The authors have nothing to report.

## Ethics Statement

This study complies with the principles of the Declaration of Helsinki. According to the policy of Plastic Surgery Hospital, Chinese Academy of Medical Sciences, and Peking Union Medical College, institutional review board approval was not required for a single de‐identified case report.

## Consent

Written informed consent for publication of the case details and clinical photographs was obtained from the patient prior to submission.

## Conflicts of Interest

The authors declare no conflicts of interest.

## Supporting information


**Supplementary Figure 1** illustrates the injection point location. The blue points on the neck represent platysma muscle relaxation points, while the red points on the masseter indicate facial slimming injection sites. The circle sizes correspond to dosage amounts.

## Data Availability

The data that support the findings of this study are available from the corresponding author upon reasonable request.
