# Supplementary figures and images for: Dexamethasone Admixture for Hypersensitivity Prevention in Botulinum Toxin Type A Treatment: A Case Report
Source: J Cosmet Dermatol. 2026 Apr 2;25(4):e70837. doi: 10.1111/jocd.70837 (PMC13044981; doi:10.1111/jocd.70837)

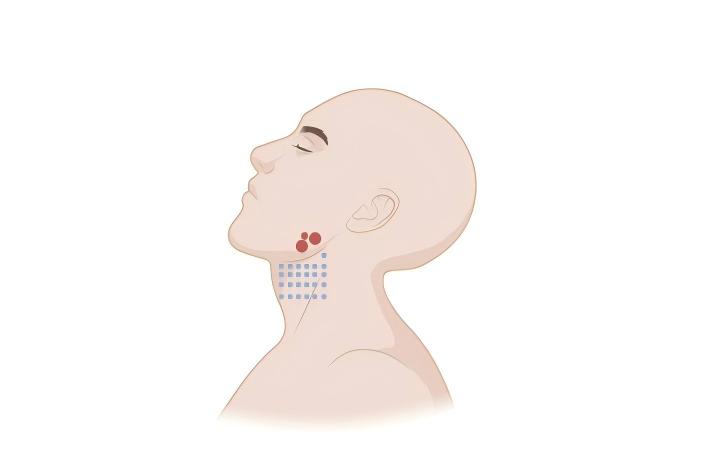

Supplement: Supplementary file 1 — Supplementary Figure 1 illustrates the injection point location. The blue points on the neck represent platysma muscle relaxation points, while the red points on the masseter indicate facial slimming injection sites. The circle sizes correspond to dosage amounts. [file JOCD-25-e70837-s001.jpg]
